# Supplementary material for: A web tool for exploring the usage of medicines in hospitals in England
Source: Wellcome Open Res. 2024 Mar 19;9:147. [Version 1] doi: 10.12688/wellcomeopenres.20766.1 (PMC11294815; doi:10.12688/wellcomeopenres.20766.1)
Supplement: Peer review history

# Reviewer report: Reviewer response for version 1

**DOI:** 10.21956/wellcomeopenres.22982.r87081

**Published:** 2024-08-01

**Authors:** Brian Mackenna

**Affiliations:** 1 University of Oxford, Oxford, England, UK

This is a succinct well written piece of work describing a web tool to explore open NHS hospital medicines data. I recommend it for indexing in its current state and thank the author for the web tool.

 I congratulate the author and I also thank the journal for providing a place to index such work. In my experience it can be difficult to find health journals that will accept descriptions of health software infrastructure.

 The paper is very clear with links to relevant code for inspection - for the avoidance of doubt, I have not fully inspected the code nor evaluated if it is enough to reproduce the tool as this would require a substantial resource investment on my part.

 The use cases in the paper give a good demonstration of the tool. I would encourage readers to actually use the tool itself to see their own examples as it is very user friendly.

 As a personal preference I would make the following observation that the author may consider revising or adding detail - however I note that there is limited/no resource for more work on this tool and paper. I’d be happy for it to be indexed in its current state and the following are minor suggestions based on my own interests and penance.

 Limitations section - I think the author overstates the limitations of the tool and mixes up perceived limitations of the underlying data with the tool. 

 Future plans - I think the direct analogue in OpenPrescribing might be https://openprescribing.net/chemical/ 

 ChatGPT - As a very new technology it would be helpful to have more detail on how ChatGPT is used in the workflow. This would help those of us trying to incorporate new technologies to learn more about its use cases. A note in the methods would probably suffice.

Are the conclusions about the tool and its performance adequately supported by the findings presented in the article?

Yes

Is the rationale for developing the new software tool clearly explained?

Yes

Is the description of the software tool technically sound?

Yes

Are sufficient details of the code, methods and analysis (if applicable) provided to allow replication of the software development and its use by others?

Yes

Is sufficient information provided to allow interpretation of the expected output datasets and any results generated using the tool?

Yes

Reviewer Expertise:

I have experience in medicines data and health data more broadly.

I confirm that I have read this submission and believe that I have an appropriate level of expertise to confirm that it is of an acceptable scientific standard.

# Reviewer report: Reviewer response for version 1

**DOI:** 10.21956/wellcomeopenres.22982.r85658

**Published:** 2024-07-30

**Authors:** Fergus Hamilton

**Affiliations:** 1 University of Bristol, Bristol, England, UK

Apologies for my delay with this. this is a useful technical report that describes an important and useful tool. I have shared widely with clinical colleagues who have found it useful. Well done!

 I have minor comments, only relating to minor clinical details:

- Piperacillin is always prescribed in the UK as part of the drug combination piperacillin-tazobactam and is a broad-spectrum antibiotic used in severe infection, this comment should ideally be added.
- *Streptococcus* Group A is generally called Group A *Streptococcus *(or *S.pyogenes*).

Once again, thanks for this.

Are the conclusions about the tool and its performance adequately supported by the findings presented in the article?

Yes

Is the rationale for developing the new software tool clearly explained?

Yes

Is the description of the software tool technically sound?

Yes

Are sufficient details of the code, methods and analysis (if applicable) provided to allow replication of the software development and its use by others?

Yes

Is sufficient information provided to allow interpretation of the expected output datasets and any results generated using the tool?

Yes

Reviewer Expertise:

infection, genomics, data

I confirm that I have read this submission and believe that I have an appropriate level of expertise to confirm that it is of an acceptable scientific standard.

# Reviewer report: Reviewer response for version 1

**DOI:** 10.21956/wellcomeopenres.22982.r85659

**Published:** 2024-07-05

**Authors:** Kishore Kumar Jagadeesan

**Affiliations:** 1 University of Bath, Bath, England, UK

Theo Sanderson's article introduces a web tool for analyzing secondary care medicine usage data, which is a great initiative for improving data access in healthcare. However, there are several critical areas where the article needs significant improvement:

**Purpose of the Tool: **The article says the tool is designed to make secondary care medicine usage data more accessible. However, it should better explain the specific problems it solves, such as handling complex raw data and providing a user-friendly interface that current tools lack.

**Documentation and Setup: **The article includes a link to the source code, which is good. However, it doesn’t provide detailed instructions or documentation on how to set up and use the tool. Without these, it’s hard for others to replicate or use the tool with different data.

**Explanation of Methods: **The article gives examples of how the tool works but doesn’t explain the statistical methods used in detail. It’s important to understand these methods to trust the results. Additionally, there should be a discussion on how the tool deals with data biases.

**Tool Performance: **The article’s conclusions about the tool’s performance are based on a few examples, which are not enough. A systematic evaluation of how accurate, user-friendly, and scalable the tool is would provide stronger support for its effectiveness.

 In summary, while the tool has great potential, the article needs significant improvements in technical details, documentation, and evaluation to meet professional standards. The author should take a more thorough approach to validate the tool’s performance and its impact on healthcare data analysis.

**Additional Citation:**

 For further information on similar tools and methodologies, you might consider looking at the PrAna R package, which is designed to aggregate and visualize England's national prescription data, focusing on primary care prescriptions. PrAna provides a comprehensive workflow for data preparation, conversion, and visualization, and offers detailed documentation for setup and use. More information about PrAna can be found in their publication and on their GitHub page (Jagadeesan *et al.*, 2022).

Are the conclusions about the tool and its performance adequately supported by the findings presented in the article?

No

Is the rationale for developing the new software tool clearly explained?

Partly

Is the description of the software tool technically sound?

No

Are sufficient details of the code, methods and analysis (if applicable) provided to allow replication of the software development and its use by others?

Partly

Is sufficient information provided to allow interpretation of the expected output datasets and any results generated using the tool?

Partly

Reviewer Expertise:

Wastewater based epidemiology,  England Primary and Secondary care Prescription Data, Data Analysis and Visualisation, Research Software Engineering, R

I confirm that I have read this submission and believe that I have an appropriate level of expertise to confirm that it is of an acceptable scientific standard, however I have significant reservations, as outlined above.

## References

[1] : PrAna: an R package to calculate and visualize England NHS primary care prescribing data. *BMC Med Inform Decis Mak* .2022;22(1) : 10.1186/s12911-021-01727-z 5 10.1186/s12911-021-01727-z 34991567 PMC8734375
